# The First Nathan Lewis Hatfield Prize for Original Research in Medicine

**Published:** 1898-06

**Authors:** J. C. Wilson

**Affiliations:** Chairman, College of Physicians, 219 South Thirteenth Street, Philadelphia, Pa.


THE FIRST NATHAN LEWIS HATFIELD PRIZE FOR ORIGINAL RESEARCH IN MEDICINE.—The College of Physicians of Philadelphia announces through its committee that the sum of five hundred dollars will be awarded to the author of the best essay in competition for the above prize.

Subject—"A Pathological and Clinical Study of the Thymus Gland and Its Relations."

Essays must be submitted on or before January 1, 1900.

Each essay must be typewritten, designated by a motto or device, and accompanied by a sealed envelope bearing the same motto or device and containing the name and address of the author. No envelope will be opened except that which accompanies the successful essay.

The committee will return the unsuccessful essays if reclaimed by their respective writers or their agents within one year.

The committee reserve the right not to make an award if no essay submitted is considered worthy of the prize.

The treatment of the subject must, in accordance with the conditions of the trust, embody original observations or researches, or original deductions.

The competition shall be open to members of the medical profession and men of science in the United States.

The original of the successful essay shall become the property of the College of Physicians.

The trustees shall have full control of the publication of the memorial essay. It shall be published in the Transactions of the College, and also when expedient as a separate issue.

Address J. C. WILSON, M.D., Chairman,
College of Physicians,
219 South Thirteenth Street, Philadelphia, Pa.

